# Strategic science communication as planned behavior: Understanding scientists’ willingness to choose specific tactics

**DOI:** 10.1371/journal.pone.0224039

**Published:** 2019-10-22

**Authors:** John C. Besley, Kathryn O’Hara, Anthony Dudo

**Affiliations:** 1 Department of Advertising and Public Relations, Michigan State University, East Lansing, Michigan, United States of America; 2 School of Journalism and Communication, Carleton University, Ottawa, Ontario, Canada; 3 Stan Richards School of Advertising and Public Relations, University of Texas at Austin, Austin, Texas, United States of America; Middlesex University, UNITED KINGDOM

## Abstract

Strategic science communicators need to select tactics that can help them achieve both their short-term communication objectives and long-term behavioral goals. However, little previous research has sought to develop theory aimed at understanding what makes it more likely that a communicator will prioritize specific communication tactics. The current study aims to advance the development of a theory of strategic science communication as planned behavior based on the Integrated Behavioral Model. It does so in the context of exploring Canadian scientists’ self-reported willingness to prioritize six different tactics as a function of attitudinal, normative, and efficacy beliefs. The results suggest that scientists’ beliefs about ethicality, norms, response efficacy, and self-efficacy, are all meaningful predictors of willingness to prioritize specific tactics. Differences between scientists in terms of demographics and related variables provide only limited benefit in predicting such willingness.

## Introduction

A growing number of individuals and groups in the United States [1] and elsewhere [2] are interested in increasing the number of scientists who communicate and the quality of that communication. Leaders of the scientific community, in particular, are calling on their colleagues to find ways to engage their fellow citizens in meaningful discussion [3–5]. In the U.S., part of the motivation for encouraging more communication appears to be a desire to ensure that Americans continue to support funding for a broad range of scientific research topics and value the use of scientific evidence in decision-making. The expectation appears to be that more high-quality communication can “reinforce positive attitudes toward science and the scientific process”([3], p. 2)

While those who study science communication have long debated the practical and conceptual challenges of trying to foster support for science [6], the apparent opportunity to help change the science communication landscape has led to a number of studies focused on understanding what factors are associated with scientists overall willingness to communicate (for reviews, see: [7–9]) and, more recently, the types of communication objectives and the comfort level of scientists with these various objectives [10–12]. A key finding of this emerging literature is that scientists are more likely to say they will communicate in specific ways when they think their behavior will make a difference (i.e., response efficacy). There does not seem to be substantial evidence that scientists worry about what their colleagues think [10–12], despite popular concerns that scientists worry that their colleagues will look down on those who take part in public communication [13].

The current study attempts to build on this past work by focusing on trying to understand what makes scientists more (or less) willing to prioritize specific communication tactics, rather than objectives or goals. It does so by conceptualizing scientists’ choice to prioritize tactics as a type of behavior in order to build on well-established behavior change theory. Doing so is also meant to expand upon past work focused on understanding scientists’ choices about communication objectives [10, 12]. Tactics, in this regard, can be understood in the context of the strategic communication literature where they are viewed as the basic set of choices that communicators make when trying to achieve their communication objectives [14]. If ‘tactics’ sounds too mercenary, then one could substitute other terms such as “tools” or “techniques,” but this seems less precise because, as described below, not all tactics are tools or techniques.

There are many tactical choices available to communicators. Fundamental choices include selecting from a range of channels to connect with a target audience; deciding what tools to use to best access or benefit from these channels (e.g., pitches, press releases, speeches, various types of event formats); determining the content of the messages (e.g., messages of risks, benefits, overarching ways of framing an issue, how much to use narratives structures); and choosing how to deliver messages (e.g., speaker characteristics, tone). Tactics might also include choices about how to prioritize resources before, during, and after communication to enable things such as audience research and evaluation. According to this way of thinking, truly strategic communicators need to decide on overarching goals for specific audiences (e.g., obtaining tacit or explicit support for a policy, or individual behaviors such as purchasing, voting, recycling, etc.) and then draw on explicit or implicit theory to identify objectives (e.g., changes in knowledge, interest, various trust-related beliefs, beliefs about efficacy or norms, etc.) that might be expected to lead to the desired goal.

We focus on tactics in this study because, while objectives and goals are clearly important [15], those involved with training scientists to improve their communication efforts ultimately want scientists to use tactics that empirical evidence suggests are most likely to work. A focus on what it would take to get scientists to make better tactical choices is also consistent with past research that suggests that science communication trainers put a lot of focus on teaching specific tactical skills (i.e., avoid jargon, body language, etc.) and less time focusing on conceptual issues related to theory [11]. Tactical skills, in this regard, seem more concrete than communication objectives such that it might possible to increase the likelihood that a communicator will prioritize a tactic (e.g., creating opportunities to meaningfully hear from stakeholders) without having to fully articulate the overall strategy driving why the tactic might be useful in achieving desired objectives. This would require ethical discussions but it seems possible to imagine cases where a communicator could responsibly help enact a strategy without fully understanding it. Indeed, it seems unlikely that everyone involved in complex communication campaigns could be expected to fully understand the underlying strategy. In a public relations context, for example, one indicator of quality is the degree to which communication leaders think strategically; not that everyone doing communication needs to be a strategist [16]. The current focus on tactics also simply reflects a desire to see if ideas developed in the context of understanding scientists’ views about communication objectives can be adapted to trying to understand scientists’ views about communication tactics.

The six specific tactics in the current study address scientists’ willingness (1) to dress in ways that help connect with audiences, (2) to explicitly talk or write about one’s motivation to conduct research, (3) to use stories to help connect with audiences, (4) to ensure that procedures are in place so that audiences feel heard, (5) to work with fellow scientists to develop and deliver a common message and (6) to take various steps to question the credibility of opponents. The rationale for choosing these specific tactics is addressed below. These tactics are somewhat specific yet reflect our (the authors’) understanding of a range of the type of tactics that are often discussed by people such as trainers coupled with our desire to ask about a range of different choices that scientists might consider. The point was not to come up with an exhaustive list of tactics to test but, rather, a working list that includes tactics requiring a range of effort and that might be expected to have impacts on a range of potential communication objectives. Other example ‘tactics’ that we might have asked about include questions about willingness to devote resources (i.e., time or money) to audience research, presentation design elements, participant experience (e.g., childcare, food and beverages), and the choice of communicator or channel. The rationale for each tactic included in the study is discussed in turn.

- The first tactic, dressing to connect with audiences, was included because of the attention that scientists have received for personal style (e.g., [17]), the public discussions around style choices by politicians as part of communicating to connect with stakeholders (e.g., [18]), the challenges that many scientists seem to have when it comes to dressing appropriately [19], and the central role that style plays in science stereotypes [20]. The expectation is that style choices could help achieve communication objectives related to shared identity [21] and that it would not be particularly onerous for most communicators.
- The second tactic, having scientists share the pro-social motivation behind research, was included because of the role a perception of caring (i.e., warmth) plays in how people view scientists amid concerns that scientists may not be seen as adequately warm [22]. Trainers also commonly encourage scientists to open up about their motivations when talking about their research (e.g., [23]).
- The third tactic included in the study addresses both the training (e.g., [23, 24]) and substantial academic interest in storytelling (e.g., [25]). The emphasis on narrative not only reflects the ability of stories to help achieve objectives related to human interest and learning, storytelling also affords the opportunity to present compelling characters who overcome struggles to achieve pro-social gains. This tactic could be somewhat challenging to implement as it likely requires substantial skill to do effectively, both in terms of implementation and in terms of identifying appropriate stories.
- The fourth tactic also relates to the centrality that some training groups (e.g., [26]) and related academics (e.g., [2, 27]) place on the role of meaningful two-way dialogue, addressing a need to consider a perceived sense of being listened to as an objective of high-quality communication (e.g., [12]). This tactic could also involve a substantial amount of effort both in terms of planning and implementation but there all also relatively easy steps communicators can do to communicate a willingness to listen.
- The fifth tactic addressed in the study reflects the potential value that strategic communicators put on working together to define and deliver impactful and consistent messages that resonate with desired audiences (e.g., [28–30]). This value also reflects academic discussions on similar topics both in terms of how issues are framed (e.g., [31]) and what to emphasize in a campaign (e.g., risks/benefit beliefs, normative beliefs, or efficacy beliefs, in the context of the TPB) [32]. The challenge with this tactic for scientists is that it requires coordination. Again, this one involves substantial potential effort.
- Finally, the sixth tactic is meant to address a potentially detrimental tendency for some scientists to act in ways that can be construed as uncivil or aggressive efforts (e.g., [33–35]) to attack the credibility or character of those with whom they disagree, despite a lack of evidence that such tactics are unhelpful [36]. This tactic does not inherently require substantial effort except inasmuch as it may be hard for some people not to behave aggressively in certain contexts.

The literature review below will delve further into the current place of tactical thinking in the communication literature, including content meant for communicators and communication researchers. It will then transition to describing this initial study, its results, and implications.

The text below will also use the terms ‘science communication’ and ‘public engagement.’ It is, therefore, important to explain how we differentiate between the two concepts. We see science communication as something members of the science community frequently do as part of activities meant to engage various publics or stakeholders. In this regard, building on the public deliberation and opinion quality literature [37], we understand the use of the term ‘engagement’ to reflect a desire by science communicators to foster the type of higher-level “elaborated” [38], “systematic” [39], or “type 2” [40] thinking that is typically associated with the development of more stable attitudes based on a series of consistent beliefs. From this perspective, the breadth of activities [41] that use the term ‘engagement’ makes sense inasmuch as these activities typically put an emphasis on communication that is meant to be interesting to specific groups, involves meaningful dialogue, or provides compelling narratives. What these types of tactics seem to have in common is that they might all try to help people overcome a natural tendency toward “peripheral,” “heuristic,” or “type 1” thinking. Further, we believe that the range of beliefs that might develop as a function of engagement is not limited to beliefs related to facts or to the process of science as studied by, say, a literacy test. Rather, we see engagement as potentially leading to a whole host of different, new, or reshaped beliefs, as well as effects on emotions, that may be associated with a wide range of objectives. This includes, beliefs about risks and benefits; beliefs about the people involved in science (e.g., their warmth, integrity, competence, identity, etc.); beliefs about others (i.e., norms); and beliefs about citizens’ own ability to make a difference (i.e., efficacy beliefs). For these reasons, this study does not focus specifically on “public engagement” but does use the term engagement in the context of specific activities where there might reasonably be an opportunity to foster deeper-level thinking and ‘science communication’ as any instance where there is an opportunity for someone to receive or share science-related content or where survey questions to respondents used the term engagement. This means for example that we talk about communication tactics because some tactics might be expected to foster deeper-level cognitive engagement whereas others might not. Similarly, we talk about communication trainers because they can teach people both to engage people or to reach people more heuristically. In contrast, we write about scientists’ engagement activity willingness and past behavior based on the fact that the underlying survey asked about engagement activities because of our interest in substantive communication efforts meant to allow people to develop substantive beliefs about science and scientists. Science communication can also be understood as the broader sub-field within which we study such topics.

## Literature review

### Criterion and predictor variables

The fundamental insight of the current line of work is the idea that researchers can treat science communicators’ *choices* about whether to prioritize specific goals, objectives, and tactics as planned behaviors. Those who study risk communication similarly conceptualized information seeking as a behavior [42] and this helped lead to the development of the Risk Information-Seeking and Process Model and its recognition that information seeking can be understood as a planned behavior driven by normative and efficacy beliefs related to the underlying topic. Conceptualizing science communicators’ choices about goals, objectives, and tactics similarly means that people who want to shape science communicators’ behaviors can draw on behavior change theories. This is important to communication researchers such as ourselves who want to find ways to get more science communicators to make evidence-based communication choices, including smart choices about when to use specific tactics.

There is a long history of behavior-change research and the current approach thus largely seeks to build on the Integrated Behavioral Model (IBM) [43] and the related Theory of Planned Behavior (TPB) [32], as these approaches have demonstrated their utility in helping to understand and influence behaviors over which people can have purposeful control. These theoretical frameworks—and their focus on attitudes, norms, and efficacy as the most important statistical primary predictors of behavioral intent—have also been the basis for many of the most recent attempts to understand scientists’ communication choices. Besley and Dudo [9] therefore argued that it makes sense to work towards a theory of strategic science communication as planned behavior. As with this past research, we chose to build on the IBM (which itself is built largely on the TPB, as well as a host of theories using similar constructs) because of both our conceptualization of tactical prioritization as something that a strategic communicator must do with intent (i.e., it is ‘planned’ behavior) and because the IBM is explicitly designed to integrate the primary variables (i.e., beliefs related to attitudes, norms, and efficacy) that past research has shown can drive such behavioral intention, a primary precursor of a planned behavior. Specifically, the current study looks at the relationships between scientists’ willingness to use specific tactics and five variables from this emerging theory of strategic science communication alongside several control variables. This approach is consistent with recent research seeking to understand the correlates of scientists’ prioritization of various science communication objectives for face-to-face [12] and online [10] engagement activities.

It looks at the relationships between scientists’ willingness to use the six tactics described above as a simple linear function of the degree to which each scientist believes that a tactic is ethical, the degree to which they believe that their colleagues would themselves use or approve of a tactic, and the degree to which they believe they are able to use a tactic and that the tactic is likely to be effective. The ethicality question is understood to represent an attitudinal belief in the context of the IBM. Further, the questions about colleagues’ views represent what the IBM would term descriptive and injunctive normative beliefs, and the questions about ability and effectiveness represent self-efficacy and response-efficacy. We also include a measure of whether the respondent has previously considered the tactic as a self-report measure of knowledge about the behavior. Whereas a typical IBM or TPB study focuses on intent to perform a behavior, willingness is used as the criterion or outcome variable in the current project because it did not make sense to ask scientists about intent to perform a behavior in the absence of a specific context. Further, Fishbein and Jazen ([32], pp. 42–43) argue that willingness is conceptually close enough to behavioral intent to make sense for some projects. The recent work on scientists’ communication objectives also focused on their willingness to prioritize certain objectives. One important difference between the current study and the TPB is that, as with the IBM, we take a direct measurement approach to attitudes, norms, and self-efficacy beliefs rather than an expectancy value approach. Practically, this means we only ask directly about evaluative beliefs and do not multiply these believes by an evaluation of these beliefs (i.e., how important is being ethical to the respondent, or motivation to comply with social norms [32]). It is partly for this reason that the study explicitly describes the predictor variables as attitudinal, normative, efficacy *beliefs* throughout. Also important to recognize is that the IBM and the TPB are sometimes used with longitudinal data where initial attitudinal beliefs, normative beliefs, and efficacy beliefs are used to predict a later behavior. This is not done in the current context as the research is still at the point of trying to identify key beliefs that people like communication trainers might use to try to shape scientists’ use of specific of desirable communication tactics.

The well-known nature of the IBM/TPB predictors makes it reasonable to make at least five simple predictions related to willingness to choose specific communication tactics. These hypotheses are interesting inasmuch as different behaviors are typically associated with different patterns of predictors. For example, while normative beliefs may be useful for changing some behaviors such as environmental action (e.g., [44]), only a few projects (e.g., [45]) have found that such beliefs affect scientists’ communication-related behavior. Indeed, a primary purpose of surveys using models such as the IBM, is to identify the relative degree to which attitudes, norms, and efficacy might be associated with a desired behavior so interventions can be designed to target objectives that are most likely to be effective.

In the current case, the first hypothesis is that a positive attitudinal belief toward each tactic would be associated with a greater willingness to choose a tactic (H1). We understand an attitude as “a latent disposition or tendency to respond with some degree of favorableness or unfavorableness” ([32], p. 76) to an object, including tactical choices. As noted, the perceived ethicality of each tactic was chosen as the focus of the attitude question because it was expected that scientists’ evaluation of whether or not a tactic was morally acceptable seemed likely to be an important evaluative belief underlying views about the tactic, similar to findings in past work on scientists’ prioritization of specific objectives (e.g., [10]).

Next, we anticipated that scientists’ willingness to use a tactic would increase with their beliefs about both what their colleagues do (descriptive norms) and view as acceptable (injunctive or subjective norms). ‘Norms’ are understood as beliefs that foster social pressure to perform or not perform behaviors and are part of a broad range of models meant to predict behavior. These two norm variants were initially expected to have separate hypotheses but, as is discussed in the method section, high correlations between the normative belief questions, it made more sense to assess a single norm-related hypothesis than those who believe that their colleagues endorse a tactic are more likely to use that tactic (H2). Past work on objectives does not support this hypothesis [10–12] but there studies seeking to predict overall engagement have sometimes found that norms matter [45]. Further, it seems reasonable to think that scientists might be more attuned to what their colleagues think about specific tactics than what their colleagues think about objectives. Tactics are more visible (i.e., you can see if someone dresses more nicely than usual) and the past work on objectives suggested that most scientists had not previously thought about most objectives. This makes it possible that any impact of normative beliefs was likely to be weak or attenuated by measurement error. Also, there are clearly many other issues where research has found that normative beliefs affect behavioral choices [46].

For efficacy, one would also expect that scientists might be more willing to consider a tactic if they believe that the tactic would be effective (response efficacy) (H3) and that they had the skill to achieve that objective (self-efficacy) (H4), similar to past work on objectives [10]. One important note for efficacy, however, is that the TPB and IBM includes efficacy in the context of personal agency, whether as a ‘control belief’ related to whether a respondent feels they are able to enact a behavior or as a measure of ability (i.e. self-efficacy). Response efficacy was included here, even though it is not part the TPB or IMB because it makes sense to us that a scientist would only be willing to use a tactic they think is likely to be effective. This is consistent with past studies by political communication scholars interested in ‘external efficacy’ (e.g., [47]), as well as health (e.g., [48]) and environmental communication (e.g., [49]) research related to response efficacy where there is an interest in knowing if people think a behavior will help solve a relevant problem. Response efficacy, in this regard, is central to widely used theories such as Protection Motivation Theory [50] and Social Cognitive Theory (in the form of ‘outcome expectancies’) [51]. Conceptually, response efficacy beliefs might also be understood as attitudinal or risk/benefit belief about a behavior but we prefer to use the term response efficacy because of its focus on the perceived effectiveness of potential choices.

A final hypothesis made for the current study is that scientists will be more willing to consider a tactic if they have thought about that tactic (H5), a concept that might be understood as similar to what the IBM describes as knowledge about a behavior or perhaps the salience of the behavior ([43], p. 77), although its inclusion in the current study also reflects a simple interest in understanding whether consideration or familiarity with a tactic makes a tactic more likely. Salience, in this regard, should be understood as the degree to which the behavior is likely to be ‘top-of-mind’ and thus more likely to be considered. The current study does not directly seek to include IBM elements such as actual or perceived constraints on behaviors or habit, although our operationalization of self-efficacy conceptually overlaps with such concepts.

The measurement of the predictor and criterion variables is described in the method section, including a justification for the use of single-item measures for key constructs.

### Additional control variables

A number of variables were included as controls in the current study. These represent the types of additional contextual variables that are often included in IBM or TPB studies but are not of substantial theoretical interest because they are not the types of factors that communication training interventions can change (e.g., gender, age, personal background). These are described in the method section.

Three types of contextual variables were ultimately excluded from the models for parsimony. These included variables aimed at assessing the Canadian scientists’ academic field, geographic location, and language. Various permutations of these variables were included in an initial set of models but they provided no statistical benefit (but took up excess space in the reporting of the results) and were subsequently excluded from the models presented.

## Methods

### Sample and implementation

The population for the project consisted of scientists from 20 Canadian research universities who were listed in the Natural Science and Engineering Research Council of Canada (NSERC) recipient database as having received a “Discovery Grant” between 2012 and 2017. Discovery grants are a standard award that Canadian academic scientists use to conduct their research. The universities were selected either because they were among the largest research universities or to ensure that each province was represented. Two research assistants manually searched online websites for email addresses for respondents in the database. The initial population included 6,984 email addresses of which 214 were returned as undeliverable. In the end, after four emails [52] between December 2017 and January 2018 (with a break over the Christmas holidays), 1,141 scientists completed the survey for a response rate of 17 percent. This was slightly better than similar American projects that used email surveys with response rates closer to 10 percent (e.g., [10, 12]), though still lower that some other projects that have used hardcopy mail surveys (e.g., [53]). It should be noted that, while the sample involved an attempted census, we still treat it as a sample in the analyses below, although the primary sources of error would be nonresponse and measurement error, rather than sampling error. More importantly, the focus here is on the relationships between the variables and not on making point estimates related to scientists’ views about engagement activities.

Respondents started with questions about overall past engagement and willingness to engage followed by questions related to views about engagement overall. The survey then transitioned to questions about tactics. To avoid survey fatigue, respondents were randomly assigned a set of questions about four of seven possible tactics. These sets were presented in random order but the order of questions within the sets were consistent. The results of six of these tactics are reported here. A seventh set of tactics with questions about their willingness to ‘speak differently depending on the audience’ is not included because almost all respondents indicated they were willing to change how they talk, avoiding jargon or technical terms based on their audience. This meant there was little variance to be explained by a statistical model. The survey then ended with background questions, including demographics. The modal time to complete the survey was about 18 minutes.

**Fig 1:**
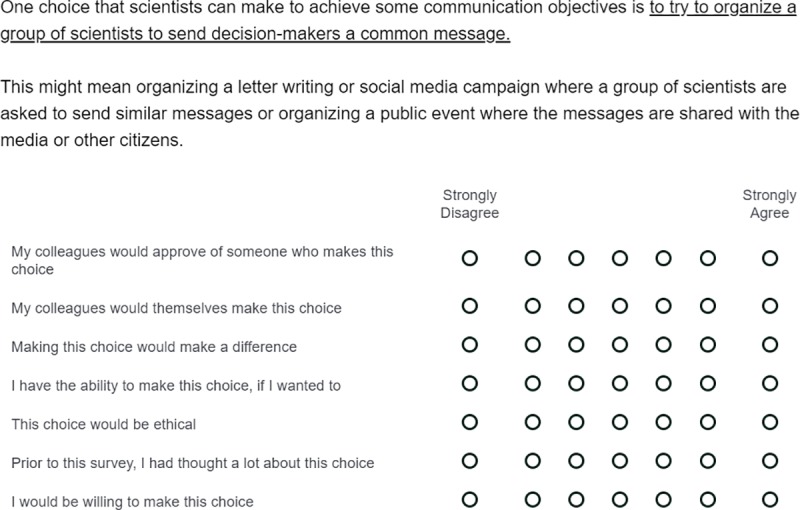
Example of question tactic-focused question block.

As discussed above, respondents could be asked about these six tactics. The reported text is as follows and an example layout can be seen in Fig 1.

- “… dress in a way that helps to connect with an audience. This might mean wearing more or less formal clothing than normal.”
- “… tell first person stories in a way that helps to connect with an audience. This might mean spending less time talking about scientific findings to have more time for providing a clear, compelling narrative about why you study your topic, your research choices, the challenges you faced and how you overcame them.”
- “… talk about the role a desire to help their community or society plays in shaping their research. This might mean spending less time talking about scientific findings to have time to talk about why you chose a science career or what you hope to achieve through your science.”
- “…make sure that nonscientists feel like they are being listened to by the scientific community. This might mean spending less time talking about scientific findings to have more time for discussion, questions and comments.”
- “… try to organize a group of scientists to send decision-makers a common message. This might mean organizing a letter writing or social media campaign where a group of scientists are asked to send similar messages or organizing a public event where the messages are shared with the media or other citizens.”
- “… publicly question the credibility of those who disagree with a scientific consensus. This might mean describing such people as deniers, liars, anti-science, or otherwise criticizing their motives or knowledge.”

### Measurement

One important limitation of the current study is that many of the central variables were measured using single-items rather than multi-item response scales, which can be helpful in managing measurement error. The decision to use single-item measures was made for practical purposes, mainly to limit respondent burden associated with long surveys. Also, given the audience and the relatively straight-forward nature of the questions, it seemed reasonable to expect that single-item questions would work for the current study. Of course, as discussed below, additional, follow-up research focused on specific tactics might benefit from using more multi-item measures.

### Criterion variables

The criterion variables for the six models were the last questions asked in each of the six tactics-focused response blocks (see Fig 1). Specifically, the question asked respondents to indicate the degree to which they agreed or disagreed that they “would be willing to make this choice.” Means and standard deviations for these questions, by tactic, are provided in Table 1.

**Table 1:** Descriptive statistics for all variables used in analyses by tactic model.

	Tactic: Dress to “help connect with an audience”		Tactic: Talk about how “a desire to help” shapes research		Tactic: Tell “stories” to help connect with audiences		Tactic: Ensure stakeholders feel “listened to”		Tactic: Organize to send “… a common message”		Tactic: Attack “the credibility” of opponents of science	
	M	SD	M	SD	M	SD	M	SD	M	SD	M	SD
***Criterion Variables and Range***												
Tactic Willingness (1–7)	5.17	1.58	5.16	1.52	5.49	1.46	5.23	1.52	4.92	1.65	4.08	2.00
***Block 1 Predictors and Ranges***												
Age (29–88)	51.31	10.69	51.37	10.69	51.60	10.96	52.06	10.54	51.95	10.71	51.73	10.63
Male (0–1)	0.71	0.46	0.72	0.45	0.74	0.44	0.71	0.45	0.71	0.45	0.72	0.45
White (0–1)	0.85	0.36	0.86	0.35	0.85	0.36	0.86	0.34	0.86	0.35	0.83	0.38
Liberal (1–7)	5.57	1.28	5.51	1.26	5.57	1.20	5.62	1.22	5.55	1.24	5.56	1.28
Perceived Impact (1–7)	5.28	1.54	5.24	1.48	5.34	1.53	5.28	1.53	5.31	1.50	5.30	1.50
Field is controversial (1–7)	3.05	1.95	2.92	1.95	3.04	1.97	2.93	1.90	3.01	1.99	3.06	1.97
Past Training (1–7)	2.15	1.44	2.11	1.41	2.13	1.46	2.22	1.49	2.14	1.46	2.15	1.44
New Media Use (1–7)	3.03	1.42	2.91	1.36	2.92	1.30	2.97	1.43	2.93	1.37	2.96	1.40
Traditional Media Use (1–7)	3.73	1.25	3.72	1.26	3.69	1.25	3.78	1.30	3.69	1.24	3.71	1.28
Past Engagement (1–7)	2.18	1.03	2.18	1.06	2.14	1.05	2.24	1.09	2.18	1.07	2.15	1.06
Engagement Willingness (1–7)	5.25	1.24	5.18	1.29	5.19	1.32	5.21	1.30	5.11	1.34	5.15	1.32
***Block 2 Predictors and Ranges***												
Ethicality Beliefs (1–7)	4.97	1.69	5.59	1.28	5.55	1.37	5.63	1.31	5.75	1.19	4.66	1.87
Normative Beliefs (1–7)	4.75	1.30	4.93	1.26	4.96	1.25	4.80	1.20	5.12	1.24	4.38	1.50
Response Efficacy Beliefs (1–7)	4.23	1.50	4.96	1.25	5.27	1.28	5.07	1.41	5.00	1.39	4.17	1.70
Self-Efficacy Beliefs (1–7)	5.88	1.19	5.62	1.27	5.73	1.30	5.58	1.28	5.71	1.23	5.35	1.59
Prior Consideration (1–7)	3.27	1.93	3.83	1.87	4.06	1.96	3.73	1.91	3.94	1.94	3.62	1.99
*Sample size (n)*		*550*		*533*		*581*		*547*		*570*		*560*

Notes: M = Mean and SD = Standard deviation. All criterion and block 2 variables measured using 7-point strongly disagree to strongly agree scales. See main text for measurement of other variables. Range in parentheses. Respondents were randomly assigned to answer questions about four tactics (overall N = 1,140).

#### Predictor variables

As noted, the primary predictor variables in the current study come from the Integrated Behavioral Model and address both normative beliefs and efficacy beliefs, as well as attitudinal beliefs in the form of perceived ethicality beliefs. The attitude question (H1) focused on belief in the perceived ethicality of a tactic (“this choice would be ethical”). Normative beliefs were measured with a question about injunctive norms (“my colleagues would approve of someone who makes this choice”) and descriptive norms (“my colleagues would themselves make this choice”). These two questions, however, were highly correlated for all six tactics (average *r* = .75) and we therefore decided to combine them into a single measure (H2). The self-efficacy (H3) (“I have the ability to make this choice, if I wanted to”) and response efficacy (H4) (“making this choice would make a difference”) were only moderately correlated (average *r* = .41) and were therefore kept separate. A final question simply asked respondents if they had thought about each tactic before the survey (H5) (“prior to this survey, I had thought a lot about this choice”).

#### Control variables

In addition to the main predictor variables, our models included control variables that we believed might serve as predictors of willingness to consider specific tactics (i.e., the six criterion variables). These variables do not directly stem from the theory at core of this study; they instead address potential questions that may be of interest to those who train or support science communicators and are included to enable discussion of the degree to which such variables might need additional theoretical attention. Several of the variables dealt with demographics. The first of these variables was age, measured in years. On average, respondents were just above 50 years old. The rationale for this variable is that it might be expected that older and younger scientists could differ on the types of tactics they would be willing to use. Similarly, variables were included for whether the respondent identified as male or white (both dummy coded as 1). About 70 percent of respondents said they were male and 85 percent of respondents said they were white. As with age, the logic of including this variable is simply that trainers might like to know if individuals from majority or less-represented groups think differently about potential tactics. Ideology was included because it might be expected that people with different values might have different views about communication tactics. This variable was measured using a 7-point scale going from “very conservative” [1] to “very liberal” [7]. The mean is well above the scale mid-point, highlighting a relatively liberal skew of Canadian scientists. Education level was not included in the study because the focus on research scientists meant all participants were highly educated (i.e., they had a Ph.D. or the equivalent).

Several other variables beyond demographics were also included because logic and informal discussions with communication practitioners suggest that these factors might be expected to shape willingness to consider different tactics. The first of these addressed respondents’ perceived place in the field. This measured combined two 7-point measures that asked respondents to say how impactful they have been in their career from "less impact than peers” [1] to “more impact than peers” [7] and to describe their publication and grant record from “many fewer than peers” [1] to “many more than peers” [7]. In general, the respondents gave responses more than a point above the measures midpoints for both questions (*M* = 5.29, *SD* = 1.65 and *M* = 5.33, *SD* = 1.66, *n* = 1,108) and the measures were highly correlated (*r* = .70, *p* < .00). The logic of including this variable is that trainers might like to know whether the most impactful scholars (as self-evaluated) are more likely to be willing to use certain tactics. These results suggest that the sample felt that they were relatively more impactful than their peers.

Another such variable concerned communication training over their career span. Respondents could choose between “no training” [1] and “a great deal of training (e.g., multiday training and personal coaching) [7]. The reported mean is well below the scale midpoint, highlighting the fact that most respondents said they had received little to no training. This variable is included simply because one might expect some training to lead to changes in how scientists see various tactics. The next control variable addressed whether respondents felt they studied a controversial topic. In this case, respondents were simply presented with the statement that “the subject I study is controversial” and asked how much they agreed or disagreed using a 7-point scale anchored by “strongly disagree” [1] to “strongly agree” [7]. The average was about a point-below the scale midpoint suggesting most felt that their topic was noncontroversial.

Finally, four variables associated with media use and overall engagement willingness were included with the logic that those who are paying more attention to societal debates might have different views about communication tactics. In other words, although it is not our primary focus, we think it is reasonable to expect that scientists who communicate and pay attention to others’ communication through media use might have different views about the acceptability or utility of tactics than scientists who pay less attention to communication (and that should be controlled for). Further, if we were to find that these variables are substantially correlated to views about tactics but not significant predictors on their own then it might make sense to consider (in future research, for example) whether our predictor variables originate with communication activities and that our theory-based predictors mediate the effect of these activities.

The two variables on media use included one focused on new media and one focused on traditional media. Both were measured using four questions, each used 7-point scales anchored by “never” [1] to “everyday” [7]. The new media questions asked about how often the respondent used “online only news sites (e.g., Slate, Huffington Post)” (*M* = 3.50, *SD* = 2.01), “blogs, online forums, including message boards and wikis” (*M* = 2.72, *SD* = 1.68), “social networking sites (e.g., Facebook, Twitter, LinkedIn)” (*M* = 3.01, *SD* = 2.12) and “video sharing sites (e.g., YouTube)” (*M* = 2.50, *SD* = 1.40) and these were added together and divided by the number of items to form a single indicator (*M* = 2.94, *SD* = 1.37; *alpha* = .74, *n* = 1,107). The traditional media questions asked about “television and films/documentaries (including online)” (*M* = 3.12, *SD* = 1.45), “magazines (including online)” (*M* = 3.58, *SD* = 1.66), “newspapers (including online)” (*M* = 4.43, *SD* = 1.87) and “radio (including online)” (*M* = 3.70, *SD* = 1.98). These were similarly combined to create composite 7-point scale (*M* = 3.71, *SD* = 1.25; *alpha* = .68, *n* = 1,109). These means suggest somewhat moderate media use with substantial variation across the sample.

A “past engagement activities” measure was created using four additional questions asked using 7-point scales anchored “never” [1] and “once a week or more” [7] that asked respondents how often in the last year they had used different channels to attempt to engage nonscientist adults. These included “online engagement through websites, blogs and/or social networks (e.g., Facebook, Twitter)” (*M* = 2.29, *SD* = 1.83), “face-to-face engagement … (e.g., giving a public talk or doing a demonstration)” (*M* = 2.33, *SD* = 1.30), “interviews or briefings with a journalist or other media professional (e.g., from a newspaper, television, online news site, documentary film, etc.)” (*M* = 2.23, *SD* = 1.25) and “direct interaction with government policy makers (e.g., meeting with elected officials, government officials, lobbyists, etc.)” (*M* = 1.78, *SD* = 1.20). The questions were combined into a new 7-point scale (*M* = 2.16, *SD* = 1.06; *alpha* = .74, *n* = 1,119). This low mean reflects the fact that most respondents have done limited engagement activity. Future willingness to engage was measured using the same four channels for engagement (*M* = 4.06, *SD* = 2.06; *M* = 5.66, *SD* = 1.51; *M* = 5.37, *SD* = 1.62 and *M* = 5.42, *SD* = 1.61) and similarly combined (*M* = 5.13, *SD* = 1.32; *alpha* = .77, *n* = 1,138). This higher mean reflects substantial willingness to engage.

### Modeling

Most of the analyses below are based on Ordinary Least Squares (OLS) regression modeling. The unstandardized co-efficient (B) is the focus of many of the results given that primary measures of interest were all measured using identical scales. These are reported in Table 2 along with the lower and upper level 95 percent confidence intervals (LLCI and ULCI, respectively) to allow for limited comparisons between models. Correlations and standardized coefficients are provided, and a sequential OLS regression was run to obtain adjusted-*r*^2^ scores for each block. The correlations and standardized coefficients are provided to enable further discussion of the relative effect size of the underlying relationships explored (before and after controls) and the adjusted-*r*^2^ scores are provided to enable a discussion of the overall amount of variance explained by the criterion variables beyond what is explained by the controls. The model presented in Table 2 is the full model. Coefficients for the initial model where the control variables were used are not shown for parsimony. Further, only variables that could logically proceed beliefs about tactics are included in the control block. Variance Inflation Factor scores (not shown) were examined and did not suggest substantial issues with multicollinearity.

**Table 2:** Correlations and OLS regression models for willingness to choose a tactic.

	**Tactic: Dress to “help connect** **with an audience” (n = 550)**						**Tactic: Talk about how “a desire** **to help” shapes research (n = 533)**						**Tactic: Tell “stories” to help** **connect with audiences (n = 581)**					
	**r**	**B**	**95%** **LLCI**	**95%** **ULCI**	**Beta**	**Sig.**	**r**	**B**	**95%** **LLCI**	**95%** **ULCI**	**Beta**	**Sig.**	**r**	**B**	**95%** **LLCI**	**95%** **ULCI**	**Beta**	**Sig.**
Intercept		-0.87	-1.70	-0.04		.04		-1.14	-1.90	-0.39		*<* .*00*		-0.81	-1.55	-0.07		.03
Age (29–88)	.00	0.00	-0.01	0.01	0.03	.39	**-.02**	**0.01**	**0.00**	**0.02**	**0.08**	**.01**	-.12*	0.00	-0.01	0.00	-0.03	.29
Male (0–1)	-.04	-0.18	-0.38	0.02	-0.05	.07	-.12*	-0.12	-0.30	0.07	-0.03	.21	-.09*	-0.01	-0.18	0.17	0.00	.94
White (0–1)	.03	0.05	-0.19	0.30	0.01	.67	.03	-0.17	-0.40	0.06	-0.04	.15	.04	0.06	-0.15	0.27	0.01	.57
Liberal (1–7)	**-.01**	**-0.07**	**-0.14**	**0.00**	**-0.06**	**.05**	.08*	-0.03	-0.09	0.04	-0.02	.38	.12*	0.02	-0.05	0.08	0.01	.65
Perceived Impact (1–7)	.06	-0.02	-0.08	0.05	-0.02	.60	**-.02**	**-0.11**	**-0.17**	**-0.05**	**-0.11**	***<* .*00***	.10*	0.02	-0.03	0.07	0.02	.46
Field is controversial (1–7)	**.02**	**-0.05**	**-0.10**	**-0.01**	**-0.07**	**.03**	.12*	0.03	-0.01	0.07	0.04	.19	.13*	-0.01	-0.05	0.03	-0.01	.68
Past Training (1–7)	.11*	0.00	-0.07	0.06	0.00	.91	.17*	0.03	-0.03	0.09	0.03	.27	.26*	0.04	-0.01	0.10	0.04	.13
New Media Use (1–7)	.05	-0.04	-0.11	0.03	-0.03	.30	.11*	**-0.07**	**-0.14**	**0.00**	**-0.07**	**.04**	.12*	-0.02	-0.09	0.04	-0.02	.49
Traditional Media Use (1–7)	.04	0.04	-0.03	0.12	0.04	.27	.17*	0.03	-0.04	0.10	0.03	.40	.11*	-0.01	-0.08	0.06	-0.01	.75
Past Engagement (1–7)	.18*	0.08	-0.02	0.19	0.06	.12	.18*	-0.06	-0.16	0.04	-0.04	.26	.29*	-0.04	-0.13	0.05	-0.03	.43
Engagement Willingness (1–7)	.26*	0.08	0.00	0.16	0.06	.06	.39*	**0.24**	**0.16**	**0.32**	**0.21**	***<* .*00***	.43*	**0.20**	**0.13**	**0.27**	**0.18**	***<* .*00***
*Adjusted R*^*2*^					.*06*	*<* .*00*					.*20*	*<* .*00*					.*22*	*<* .*00*
Ethicality Beliefs (1–7)	.50*	**0.18**	**0.12**	**0.24**	**0.19**	***<* .*00***	.52*	**0.14**	**0.07**	**0.22**	**0.12**	***<* .*00***	.50*	**0.15**	**0.08**	**0.21**	**0.14**	***<* .*00***
Normative Beliefs (1–7)	.55*	**0.29**	**0.21**	**0.37**	**0.24**	***<* .*00***	.51*	**0.11**	**0.04**	**0.19**	**0.09**	***<* .*00***	.46*	**0.11**	**0.04**	**0.18**	**0.09**	***<* .*00***
Response Efficacy Beliefs (1–7)	.57*	**0.30**	**0.23**	**0.37**	**0.29**	***<* .*00***	.66*	**0.35**	**0.26**	**0.44**	**0.29**	***<* .*00***	.63*	**0.27**	**0.20**	**0.35**	**0.24**	***<* .*00***
Self-Efficacy Beliefs (1–7)	.52*	**0.35**	**0.27**	**0.43**	**0.26**	***<* .*00***	.65*	**0.34**	**0.26**	**0.42**	**0.28**	***<* .*00***	.67*	**0.39**	**0.32**	**0.46**	**0.35**	***<* .*00***
Prior Consideration (1–7)	.37*	**0.08**	**0.03**	**0.13**	**0.10**	***<* .*00***	.48*	**0.13**	**0.08**	**0.18**	**0.16**	***<* .*00***	.48*	**0.09**	**0.04**	**0.13**	**0.11**	***<* .*00***
*Adjusted R*^*2*^					.*59*	*<* .*00*					.*65*	*<* .*00*					.*63*	*<* .*00*
	**Tactic: Ensure stakeholders** **feel “listened to” (n = 547)**						**Tactic: Organize to send “… a** **common message” (n = 570)**						**Tactic: Attack “the credibility”** **of opponents of science (n = 560)**					
	**r**	**B**	**95%** **LLCI**	**95%** **ULCI**	**Beta**	**Sig.**	**r**	**B**	**95%** **LLCI**	**95%** **ULCI**	**Beta**	**Sig.**	**r**	**B**	**95%** **LLCI**	**95%** **ULCI**	**Beta**	**Sig.**
Intercept		-0.79	-1.58	0.00		.05		-1.96	-2.92	-1.01		.00		-1.49	-2.35	-0.63		.00
Age (29–88)	.01	0.00	-0.01	0.01	0.02	.41	-.06	0.00	-0.01	0.01	0.03	.41	.02	0.00	-0.01	0.01	-0.02	.54
Male (0–1)	-.10*	**-0.19**	**-0.37**	**-0.01**	**-0.06**	**.04**	-.10*	-0.15	-0.36	0.07	-0.04	.19	.09*	0.03	-0.20	0.26	0.01	.78
White (0–1)	.04	0.13	-0.10	0.37	0.03	.27	.07*	-0.04	-0.32	0.24	-0.01	.78	.01	-0.24	-0.50	0.02	-0.04	.08
Liberal (1–7)	.04	-0.05	-0.12	0.01	-0.04	.13	.19*	**0.08**	**0.00**	**0.16**	**0.06**	**.05**	.16*	0.07	-0.01	0.15	0.05	.07
Perceived Impact (1–7)	.02	-0.04	-0.10	0.02	-0.04	.20	.03	0.03	-0.04	0.10	0.02	.46	.11*	0.01	-0.06	0.08	0.01	.71
Field is controversial (1–7)	.14*	-0.01	-0.06	0.03	-0.02	.55	.12*	0.00	-0.05	0.05	0.00	.93	.04	0.01	-0.04	0.06	0.01	.74
Past Training (1–7)	.09*	**-0.07**	**-0.13**	**-0.02**	**-0.07**	**.01**	**.04**	**-0.09**	**-0.16**	**-0.01**	**-0.08**	**.02**	.05	0.01	-0.06	0.08	0.01	.79
New Media Use (1–7)	**.07**	**-0.08**	**-0.15**	**-0.01**	**-0.08**	**.02**	.13*	0.00	-0.09	0.08	0.00	.93	.09*	-0.06	-0.15	0.02	-0.04	.15
Traditional Media Use (1–7)	.16*	**0.12**	**0.05**	**0.19**	**0.10**	***<* .*00***	.12*	0.08	-0.01	0.16	0.06	.07	.13*	0.04	-0.05	0.13	0.02	.39
Past Engagement (1–7)	.21*	-0.01	-0.10	0.08	-0.01	.84	.20*	-0.03	-0.15	0.09	-0.02	.58	.08*	0.02	-0.10	0.14	0.01	.79
Engagement Willingness (1–7)	.45*	**0.29**	**0.21**	**0.36**	**0.25**	***<* .*00***	.33*	**0.12**	**0.03**	**0.21**	**0.10**	**.01**	.16*	**0.09**	**0.00**	**0.18**	**0.06**	**.04**
*Adjusted R*^*2*^					.23	*<* .*00*					.12	*<* .*00*					.05	*<* .*00*
Ethicality Beliefs (1–7)	.49*	**0.11**	**0.04**	**0.19**	**0.10**	***<* .*00***	.45*	**0.14**	**0.05**	**0.24**	**0.10**	***<* .*00***	.75*	**0.47**	**0.39**	**0.55**	**0.44**	***<* .*00***
Normative Beliefs (1–7)	.48*	**0.08**	**0.00**	**0.16**	**0.06**	**.05**	.51*	**0.21**	**0.12**	**0.31**	**0.16**	***<* .*00***	.65*	**0.23**	**0.13**	**0.32**	**0.17**	***<* .*00***
Response Efficacy Beliefs (1–7)	.69*	**0.41**	**0.33**	**0.48**	**0.37**	***<* .*00***	.49*	**0.22**	**0.14**	**0.30**	**0.19**	***<* .*00***	.65*	**0.22**	**0.15**	**0.30**	**0.19**	***<* .*00***
Self-Efficacy Beliefs (1–7)	.60*	**0.26**	**0.18**	**0.34**	**0.21**	***<* .*00***	.50*	**0.27**	**0.17**	**0.36**	**0.20**	***<* .*00***	.44*	0.03	-0.04	0.10	0.02	.43
Prior Consideration (1–7)	.48*	**0.09**	**0.04**	**0.14**	**0.11**	***<* .*00***	.55*	**0.27**	**0.21**	**0.32**	**0.31**	***<* .*00***	.48*	**0.18**	**0.13**	**0.24**	**0.18**	***<* .*00***
*Adjusted R*^*2*^					.*62*	*<* .*00*					.*52*	*<* .*00*					.*68*	*<* .*00*

Note

*p < 0.05 (two-tailed) for Pearson and bi-serial correlations (r); B = unstandardized regression weight, LLCI = Lower Level 95% Confidence Interval, ULCI = Upper Level 95% Confidence Interval, *Beta* = Standardized regression coefficient. Exact probability shown for regression parameter estimates (two-tailed). Adjusted-R^2^ significance represents significant F-score change from that block in a sequential regression but only the final regression model with all variables is reported. Ranges in parentheses with variable names. Bolded coefficients are significant in the final regression model at the *p* < .05 level (two-tailed).

## Results

### Control variables

Very few of the control variables were meaningfully related to willingness to choose any of the six tactics studied, especially after considering predictor variables from the IBM. For the final models, the only consistent multivariate predictor of willingness to use the tactics among the controls was overall willingness to engage. The results suggest that those who are willing to take part in engagement activities are also willing to consider a range of tactics. Past training experience and past engagement behavior also both had bivariate relationships with many of the tactics but, were generally not significant predicators after controls. Training, in this regard, was only a negative significant statistical predictor of a willingness to use listening and group organization tactics after controls. Past engagement was never a significant multivariate predictor. One way to interpret this is that engagement willingness is what matters most, not past engagement or training behaviors. Past engagement is particularly correlated with willingness (*r* = .48, *p* < .00) and post-hoc re-running of the models presented without willingness (not shown) suggests that past behavior would generally be a significant (although relatively small) predictor of tactical willingness in the initial control block (on its own) and in the final model if overall engagement willingness were not in the model. In other words, it might make sense to explore whether engagement activity willingness mediates [54] the relationship between past behavior and tactical willingness, or whether the relationship is spurious. Similarly, other variables such as training experience (which is only weakly correlated with engagement willingness, r = 18, *p* < .00) that have significant correlations with tactical willingness but are not significant in the final model could also be explored. It may also be noteworthy that a willingness to organize with other scientists was somewhat more popular with relatively liberal scientists. Overall the control variables appeared to explain between about five and 23 percent of the overall variance in tactical willingness but this is almost entirely a function of the impact of the overall engagement willingness variable. The control variables also explained substantially more variance in the models where the focus was on tactics that might require more effort (i.e., not tactics like dressing and attacking).

### Predictor variables

Unlike the control variables, the predictor variables for which we had formal hypotheses were significantly and substantively related to willingness, though the pattern appeared to vary somewhat by tactic.

Ethicality beliefs (H1) were relatively small predictors of most tactics, except the tactic associated with attacking opponents. For the ‘non-attack’ tactics, a 1-point change in perceived ethicality was associated with between about a 1/10- and 2/10-of-a-point change in willingness. However, for attack variable, this increased to around 5/10-of-a-point.

Normative beliefs (H2) were relatively small predictors for about half of the tactics and more substantive predictors for the other half. For the tactics related to talking about motivation, telling stories, and listening to others, normative beliefs were relatively small predictors with a 1-point average change being associated with between about 1/10-of-a-point change in willingness. For the tactics associated with dressing, organizing, and attacking, a 1-point change in the normative beliefs measure was associated with about 2/10-of-a-point change in tactical willingness.

The variable for scientists’ perceptions that the various tactics would make a difference—an attempt to operationalize response-efficacy—was the other predictor that was most consistently associated with willingness to use a tactic; although the relationship dropped somewhat in the case of organizing to send a common message and attacking opponents’ credibility (H3). For the first four tactics, a 1-point change in perceived response-efficacy was associated with between about a 3/10- and 4/10-of-a-point change in willingness to use a tactic. For organizing and attacking, this dropped to closer to a 2/10-of-a-point change.

Scientists’ perceptions that they had personal ability to use a tactic (i.e., self-efficacy) was also one of the more important predictors in all but the model for the tactic related to attacking opponents’ credibility (H4). For the five other tactics, a 1-point change in self-efficacy was associated with between about a 3/10- and 4/10-of-a-point change in willingness to use a tactic. However, self-efficacy was not associated with willingness to attack opponents at all.

The pattern was reversed for the final predictor related to prior consideration (H5). In this case, there appeared to be a relatively small relationship between the tactics associated with dressing, talking about motivation, and story-telling with 1-point change being associated with about 1/10-a-point change in tactical willingness. Prior consideration was associated with closer to 2/10-of-a-point in the models for willingness to organize a group and attack an opponent.

## Discussion

The current study finds that attitudinal beliefs about ethics, norms, and efficacy are usually reasonable statistical predictors of scientists’ willingness to choose the six tactics studied here. This is consistent with the idea of a theory of strategic science communication as planned behavior that Besley, Dudo, and Yuan [12] proposed in the context of scientists’ prioritization of communication objectives. As noted above, the value of treating communicators’ choices as behaviors is important because it suggests substantial opportunity for theory driven exploration of why communicators such as scientists communicate in specific ways. Knowing why people involved in areas such as science communication make specific choices further introduces the opportunity of designing interventions aimed at shaping these choices.

Indeed, the current study suggests that any person (e.g., a communication trainer) or organization (e.g., a university) that wants scientists to use—or not use—a communication tactic might expect to especially benefit from assessing whether their scientists view that tactic as ethically acceptable and acceptable to their peers. Even more important, according to the relative size of the coefficients, such an actor may want to know if the desired communicators believe they have the skills to use the tactic, and believe that the tactic is likely to be effective in achieving the communicators’ goals. If a communicator has negative beliefs about some aspect of a tactic then an effort might be made to address those beliefs. For example, one might seek to increase scientists’ sense of self-efficacy about storytelling through training. Similarly, one might seek to deflect scientists’ tendency to attack others’ credibility through discussions about the questionable practicality or ethicality of aggressive approaches. Efforts to get scientists to dress for their audience might similarly highlight the fact that, at least according to the results here (Table 1), their fellow scientists do not generally see such choices as normatively problematic (i.e., the norm is well above the scale midpoint).

A range of limitations with the current and past studies suggest many opportunities for additional research. Two of the most important of these limitations relate to measurement and the choice of tactics selected for study. For measurement, it would have been better to measure each of the core constructs in the current study using multiple measures. This was not done for practical reasons related to survey length and a desire to ask about multiple types of tactics. The well-educated nature of the sample, the relative clarity of the concepts, and the robust nature of the correlations combine to make us comfortable with the approach, but future research might benefit from more focused attention on a single tactic or a smaller set of tactics. A related limitation of this study is that the authors chose the six tactics that underlie the study based on their interactions with communication trainers and knowledge of science communication research. Besley, Dudu, and Yuan’s [12]’s past work on objectives similarly focused a researcher-selected set of potential communication objectives. What is needed is more clarity on what tactics (and objectives) are most commonly recommended by research or practitioners. Such research might include both systematic literature reviews as well as structured discussions with key informants. Further, it should be noted that research in this area has been almost entirely cross-sectional in nature. While there is beginning to be innovative, peer-reviewed work that shows that science communication can improve some skills and perceived self-efficacy [55], we do not know very much about whether training can affect choices about things such as goals, objectives, or tactics. We also do not have studies that address scientists’ goal selection (as defined above) in the context of their attitudinal beliefs, normative beliefs, and efficacy beliefs.

Finally, it should be noted the current study focuses on science communication because that is the area of interest to the authors. However, there is nothing in the current study that argues that only science communicators’ choices about goals, objectives, and tactics can be better understood through attention to attitudinal beliefs, normative beliefs, and efficacy beliefs. Future work could therefore assess the degree to which other types of communicators’ (e.g., public relations practitioners, government officials, etc.) strategic choices can be meaningfully understood and shaped through attention to such variables.
